# EEG-Based Driving Fatigue Detection Using a Two-Level Learning Hierarchy Radial Basis Function

**DOI:** 10.3389/fnbot.2021.618408

**Published:** 2021-02-11

**Authors:** Ziwu Ren, Rihui Li, Bin Chen, Hongmiao Zhang, Yuliang Ma, Chushan Wang, Ying Lin, Yingchun Zhang

**Affiliations:** ^1^Robotics and Microsystems Center, Soochow University, Suzhou, China; ^2^Department of Biomedical Engineering, University of Houston, Houston, TX, United States; ^3^College of Automation, Intelligent Control & Robotics Institute, Hangzhou Dianzi University, Hangzhou, China; ^4^Guangdong Provincial Work Injury Rehabilitation Hospital, Guangzhou, China; ^5^Department of Industrial Engineering, University of Houston, Houston, TX, United States

**Keywords:** driving fatigue detection, electroencephalography, principal component analysis, radial basis function, neural network, classification

## Abstract

Electroencephalography (EEG)-based driving fatigue detection has gained increasing attention recently due to the non-invasive, low-cost, and potable nature of the EEG technology, but it is still challenging to extract informative features from noisy EEG signals for driving fatigue detection. Radial basis function (RBF) neural network has drawn lots of attention as a promising classifier due to its linear-in-the-parameters network structure, strong non-linear approximation ability, and desired generalization property. The RBF network performance heavily relies on network parameters such as the number of the hidden nodes, number of the center vectors, width, and output weights. However, global optimization methods that directly optimize all the network parameters often result in high evaluation cost and slow convergence. To enhance the accuracy and efficiency of EEG-based driving fatigue detection model, this study aims to develop a two-level learning hierarchy RBF network (RBF-TLLH) which allows for global optimization of the key network parameters. Experimental EEG data were collected, at both fatigue and alert states, from six healthy participants in a simulated driving environment. Principal component analysis was first utilized to extract features from EEG signals, and the proposed RBF-TLLH was then employed for driving status (fatigue *vs*. alert) classification. The results demonstrated that the proposed RBF-TLLH approach achieved a better classification performance (mean accuracy: 92.71%; area under the receiver operating curve: 0.9199) compared to other widely used artificial neural networks. Moreover, only three core parameters need to be determined using the training datasets in the proposed RBF-TLLH classifier, which increases its reliability and applicability. The findings demonstrate that the proposed RBF-TLLH approach can be used as a promising framework for reliable EEG-based driving fatigue detection.

## Introduction

Driving fatigue is a typical mental and physical concern that weakens the driver's ability to control the vehicle (Li Z. et al., 2017). It not only poses a significant injury and fatality risk to the drivers but also causes injury to other road users such as passengers, motorbike users, other drivers, and pedestrians. According to the statistical data reported by the World Health Organization, more than 1.3 million people are killed in traffic accidents every year mainly due to fatigued driving (Sahayadhas et al., 2012; Li Z. et al., 2017). Therefore, it is of great importance to investigate the characteristics of driving fatigue and develop an automatic driving fatigue detection system with reliable detection performance (Li Z. et al., 2017; Sikander and Anwar, 2019).

The currently available methods for driving fatigue detection can be characterized into three categories (Sikander and Anwar, 2019): (1) psychology-based approach that generally relies on psychometric questionnaires to evaluate an individual's fatigue level (Michielsen et al., 2004), (2) video-based approach that usually monitors the behavioral and physical status of the driver, such as facial features, head position, reaction time, steering errors, lane deviation, etc. (Akerstedt et al., 2005; Hsieh and Tai, 2013), and (3) physiological approach that makes use of bio-signals associated with driving fatigue, such as electrooculography (EOG) to measure the movement of the eye (Hu and Zheng, 2009; Picot et al., 2012), electrocardiography (ECG) to detect heart rate variability (Jung et al., 2014), electroencephalography (EEG) to assess brain state (Huang et al., 2016; Ma et al., 2019, 2020), and electromyography (EMG) to measure muscle activity (Sikander and Anwar, 2019). Among them, psychological self-reported measurement is time-consuming and subjective because it relies on the driver's subjective feedbacks *via* questionnaires, which makes it infeasible and unreliable for real-time detection. Video-based approaches are vulnerable to environmental factors, such as brightness, weather, road conditions, and other factors, which could result in poor detection performance (Jimenez-Pinto and Torres-Torriti, 2012). EOG, ECG, surface EMG, and EEG have all been explored as physiological measures for driving fatigue detection, with specific advantages and disadvantages to each other (Sikander and Anwar, 2019). Electrodes have to be placed over the body surface, which makes the system intrusive in nature. For example, EOG signals are retrieved through electrodes placed near the eye, which can hinder driving. ECG can be measured in a less intrusive way, but ECG signals showed a high inter-subject variance which may lead to challenges in developing a generic driving fatigue detection system. The applicability of surface EMG in real-time driving fatigue detection is limited (Sikander and Anwar, 2019). EEG has been considered as a promising modality for driving fatigue detection, owing to its high temporal resolution, high portability, and good sensitivity to brain state (O'Hanlon and Kelley, 1977; Nguyen et al., 2019; Gao et al., 2020). In particular, EEG can be used to non-invasively measure the neuronal electrical activity from the scalp surface to provide a direct assessment of brain fatigue status (Zhao et al., 2017; Sikander and Anwar, 2019). However, EEG signal retrieval through multiple electrodes is highly susceptible to noise from external factors, and it is critical to extract informative features from noisy EEG signals for a successful driving fatigue detection application.

Neural networks have been used as promising tools in extracting informative features from EEG signals because of their massive computational parallelism which resembles the way the brain processes information (Masic and Pfurtscheller, 1993). Recently, many studies have implemented EEG-based driving fatigue detection systems using neural network techniques. Vuckovic et al. proposed a model for classifying alertness and drowsiness from EEG recordings on arbitrary healthy subjects, in which the artificial neural network (ANN) was used as an automatic classifier (Vuckovic et al., 2002). Yang et al. presented a driving fatigue classification model based on information fusion technique and dynamic neural network. The experimental results indicated that the EEG-derived features were able to detect the fatigue state of a driver (Yang et al., 2010). Moreover, Aruna et al. proposed a recurrent self-evolving fuzzy neural network method for driving fatigue detection, in which the correlation coefficient of driver attention was classified to detect driving fatigue (Aruna and Kalaivani, 2016). Chai et al. presented a three-layer feed-forward Bayesian neural network structure for the binary classification of driving fatigue, where autoregressive (AR) modeling was used as the feature extraction algorithm (Chai et al., 2017b). Besides that, Chai et al. also proposed an improved EEG-based driving fatigue classification model, where the AR model was employed for feature extraction, and the sparse-deep belief network (sparse-DBN) was employed for classification (Chai et al., 2017a). Recent studies also demonstrated the radial basis function (RBF) neural network as a promising classifier due to its linear-in-the-parameters network structure, strong non-linear approximation ability, and desired generalization property. Li *et al*. demonstrated that the radial basis function-based classification method has advantages in terms of classification accuracy for epileptic seizure classification by comparing with five other classifiers (Li Y. et al., 2017; Li et al., 2019). The RBF kernel-based support vector regression also achieved better performance in fatigue prediction compared to the other kernel functions in the study of Bose et al. (2019). The performance of the RBF network heavily relies on network parameters, which should be optimized globally for best performance. The RBF network parameters can be estimated using the existing global optimization methods (Petković et al., 2016; Aljarah et al., 2018). Unfortunately, due to a relatively large number of network parameters that need to be optimized, the existing global optimization methods show high computational cost and slow convergence and further lead to low classification accuracy and efficiency of the RBF network.

In this study, a two-level learning hierarchy RBF network (RBF-TLLH) is developed to enhance the performance of the RBF classification. In the proposed RBF-TLLH, only three key RBF network parameters need to be optimized and, as such, can be easily optimized globally and efficiently. Specifically, the RBF-TLLH is constructed by employing the ROLS+D-opt algorithm, which combines the regularized orthogonal least squares (ROLS) and the D-optimality experimental design (D-opt) at the lower level and the particle swarm optimization (PSO) at the upper level. The PSO algorithm is used to globally optimize the three core parameters of the ROLS+D-opt algorithm to enhance the classification performance. As EEG signals are usually measured with multiple channels at a high sampling rate, principal component analysis (PCA) (Hotelling, 1933) is employed to reduce the dimensionality of the original data space (Lever et al., 2017; Artoni et al., 2018) before the application of the RBF-TLLH. The performance of the proposed approach is evaluated on driving fatigue detection and compared against several widely used artificial neural networks, including the artificial neural network based on back-propagation (BP), the artificial neural network based on PSO, and the RBF network based on the ROLS+D-opt learning algorithm.

## Materials and Methods

### Study Design

**Figure 1:**
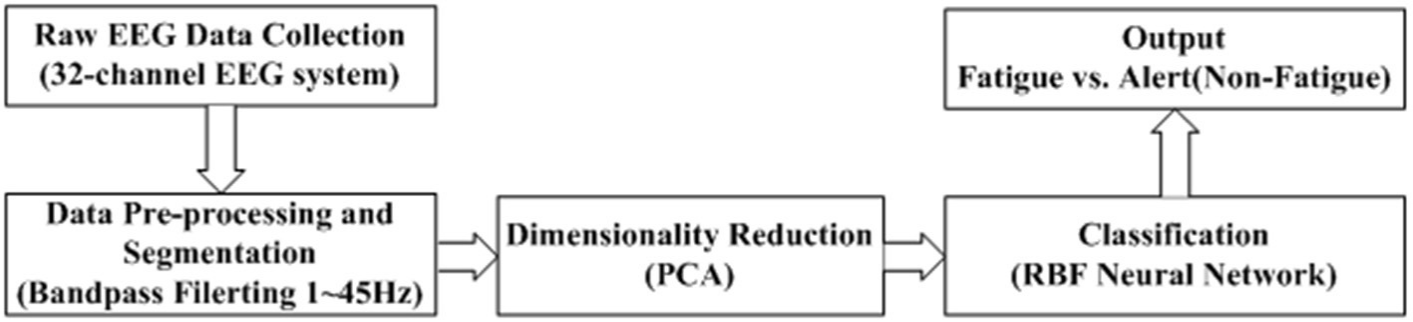
Schematic illustration of the two-level learning hierarchy radial basis function network.

The overall structure for the proposed EEG-based fatigue classification framework is shown in Figure 1, which consists of five steps: (1) EEG data collection during a simulated driving environment, (2) raw data pre-processing and segmentation, (3) dimensionality reduction and feature extraction using PCA; (4) classification using the RBF network, and (5) performance evaluation.

### Participants and EEG Data Acquisition

The EEG data used in this study were collected from six healthy, male volunteers (right-handed, aged 23 to 27 years). All volunteers had valid driver's licenses, and no participant had any history of physical or psychological disorders. The study was approved by the local ethics committee (Guangdong Provincial Work Injury Rehabilitation Center, China) and performed in accordance with the Declaration of Helsinki. Each subject was fully informed about the purpose of the research and provided written informed consent before the start of the experiment.

**Figure 2:**
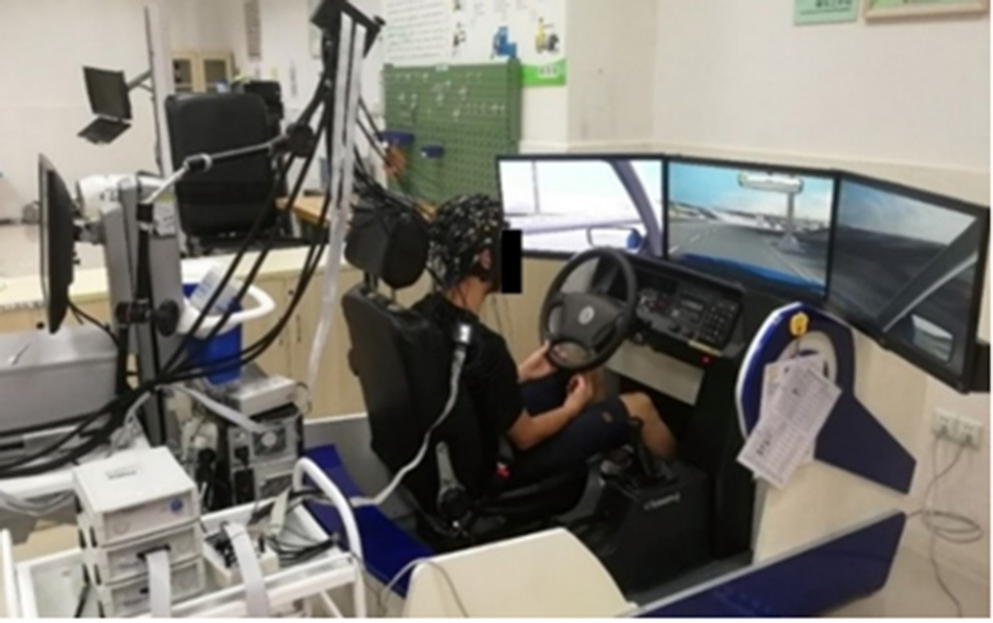
The driving simulation system and EEG acquisition device.

A driving simulation system (Shanghai Infrared Automobile Simulator Driving Equipment Co., Ltd., China) was employed to imitate a real driving environment during the experiment. As shown in Figure 2, the driving simulation system includes clutches, brakes, throttles, and scene simulations that consist of three large screens and high-performance simulation software. This system can imitate the real-driving experience, such as the changing surrounding traffic. EEG signals were recorded using a 32-channel EEG acquisition system (Brain Products GmbH, Germany), with a sampling rate of 500 Hz. EEG electrodes were placed on the scalp based on the international 10–20 standard system.

Prior to the start of the experiment, all participants were allowed to practice and get familiar with the driving simulation system. EEG data were then collected for two states, alert (non-fatigue) and fatigue. To collect the alert data, all subjects were required to maintain an adequate and natural sleep for about 8 h during the night before the experiment. The EEG data were collected at 9 a.m. on the next day for about 30–60 min while the subjects were executing the driving simulation task. For the recording of alert data, the path was set relatively complicated to avoid the drowsiness of the subjects. On the other hand, to collect the fatigue data, all subjects were requested to sleep for only 4 h during the night before the experiment. The EEG data were also recorded at 9 a.m. for 30 to 60 min when the subjects were driving in the simulation environment. The experiment was performed in a quiet and undisturbed laboratory with ambient temperatures of around 22°C. In order to reach the fatigue state rapidly in fatigue data collection, a long and straight road with very few pedestrians was used in the simulated environment. During the data recording, an observer was seated 2 m beside the subject and monitored the subject's behavior without causing any disturbance to the subject. The observer decided whether the subject was in a fatigue state or an alert state by observing the subject's drowsy signs (more than 2 s of eye closure and head nodding, large deviation off the road). The EEG data recording was terminated at a time of 30 min after the subject began to show fatigue symptoms. For the participants who stayed in alert state for 60 min, the experiments were terminated, and the participants were excluded from further analysis.

### Data Pre-processing and Segmentation

In this study, 20-min EEG signals in each state (alert or fatigue) were collected on each subject, and all the data analyses were implemented in a MATLAB environment (2014a, MathWorks, Natick, Massachusetts). The recorded EEG data were firstly down-sampled from 500 to 200 Hz, and a fourth-order Butterworth band-pass filtering (1–45 Hz) was then applied to remove artifacts such as slow drift, high-frequency noise, and power line interference. The 20-min (1,200 s) pre-processed EEG data for each state were then segmented by applying a 10-s time window, which resulted in 120 samples for each state (fatigue or alert). It is worthy of note that, in this study, each sample is a two-dimensional matrix form (32 channels × 2,000 points). As such, with the six participants, a total of 1,440 samples (720 samples for alert and 720 samples for fatigue) were formed for feature extraction and classification. For each participant, the total of 240 samples was divided into the training data set with 200 samples and the validation data set with the remaining 40 samples, where the fatigue and alert state EEG samples were evenly split. In addition, a 6-fold cross-validation was employed for performance evaluation.

### Feature Extraction

To extract the representative features from the large amounts of EEG data, dimensionality reduction is firstly performed to reduce computational expense and classification error. PCA is an efficient and flexible unsupervised method for dimensionality reduction of data (Hotelling, 1933). For a given EEG sample (*m*32-channels × 2,000 points), PCA transforms the sample data into a lower-dimension space through an orthogonal projection or transformation of the correlated points into uncorrelated variables of data, known as principal components (PCs) (Lever et al., 2017; Artoni et al., 2018). Based on the predetermined cumulative contribution rate, the first *r* components with the largest variances are preserved. The preserved number of PCs, *r*, is an important parameter in PCA. In this study, different *r*-values were tested through multiple trials, and the results showed that the first 10 PCs accounted for over 80% (the minimum cumulative contribution rate is up to 82.13%) of the total variance of the original signals for all EEG samples. Hence, the first 10 PCs were preserved, and the original EEG sample with a size of 32 by 2,000 was transformed into a lower-dimension matrix with a size of 32 by 10. These lower-dimensionality samples were used to construct the driving fatigue classification model.

### Classification Model

**Figure 3:**
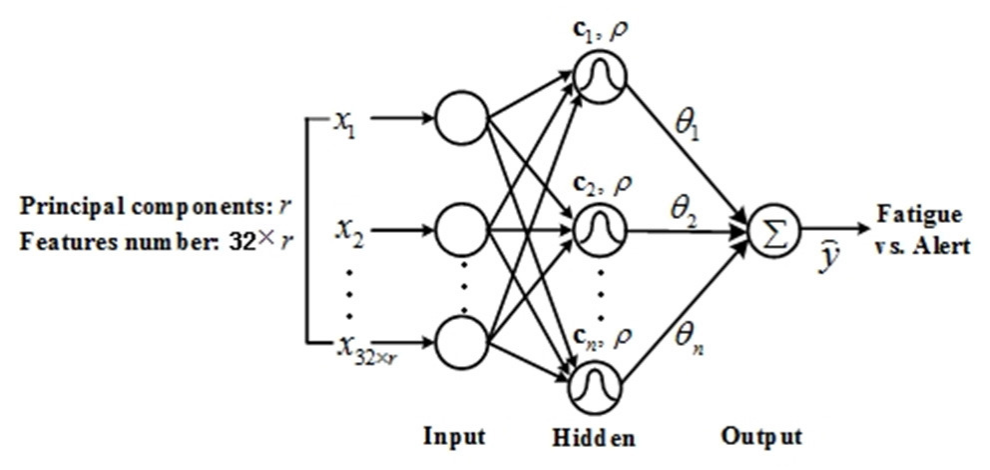
Radial basis function network for the EEG-based driving fatigue classification model.

An RBF network is a single hidden layer feedforward neural network that is generally controlled by several key parameters, including center vectors, the width of the basis function, and the connecting weights from the hidden nodes to the network output. An RBF network with *n* hidden nodes and a single output is shown in Figure 3, where the input features are first transformed to hidden nodes via *n* Gaussian basis functions with a uniform width and different center vectors. The hidden nodes are further aggregated to predict the network output *via* connecting weights. Denoting the input vector as **x** and the output as ỹ**(x)**, the RBF network could be represented as:

(1) $$\begin{array}{l} ỹ\left(\text{x}\right)=\overset{n}{\underset{i=1}{\sum }}\theta_{i}\exp\left(-{\left||\text{x}-{\text{c}}_{i}|\right|}^{2}/\rho \right) \end{array}$$

where **c**_*i*_(*i* = 1, ⋯ , *n*) are the center vectors, ρ is the width of the Gaussian basis functions, θ_*i*_(*i* = 1, ⋯ , *n*) are the weights, and ||·|| is the Euclidean norm.

#### ROLS+D-opt Integrated Learning Algorithm

In order to construct a model with good performance, in this study, we adopt an integrated learning algorithm (ROLS+D-opt) to train the model by combining regularized orthogonal least squares and D-optimality experimental design. The ROLS regularization techniques improve the generalization properties, and the D-optimality experimental design further enhances the efficiency and robustness of the model (Hong and Harris, 2002; Chen et al., 2003). Denoting the input and output of *k*th sample as **x**(*k*) and *y*(*k*), respectively, a training set of *N* samples could be represented as ${\left\{y\left(k\right),\text{x}\left(k\right)\right\}}_{k=1}^{N}$. To formulate the network as a linear-in-the-parameters problem, each sample input is considered as a candidate center in RBF, i.e., **c**_*i*_ = **x**(*i*), *i* = 1, …, *N*. Therefore, the *i*th hidden node on the *k*th sample, denoted as ϕ_*i*_(*k*), could be represented as $\phi_{i}\left(k\right)=exp\left(-\frac{||\text{x}\left(k\right)-\text{x}\left(i\right)|{|}^{2}}{\rho }\right)$. The desired output *y*(*k*) can be expressed as

(2) $$\begin{array}{l} y\left(k\right)=ỹ\left(k\right)+e\left(k\right)=\overset{N}{\underset{i=1}{\sum }}\theta_{i}\phi_{i}\left(k\right)+e\left(k\right)1\leq k\leq N \end{array}$$

where *e*(*k*) is the error between *y*(*k*) and the actual network output ỹ(*k*), θ_*i*_ are the output weights, and *N* is the number of samples in the training dataset. The integrated ROLS+D-opt learning algorithm first transforms the model (2) into a matrix form and performs orthogonal decomposition on the regression matrix, which decomposes the regression matrix to a matrix with orthogonal columns and an upper triangular matrix. Specifically, the regression model in (2) can be depicted as:

(3) $$\begin{array}{l} \text{y}=\Phi \theta +\text{e}=\text{W}\text{A}\theta +\text{e}=\text{W}\text{g}+\text{e} \end{array}$$

where **y** is output vector, **Φ** is regression matrix, **θ** is weighting vector, and **e** is the error vector. The regression matrix ****Φ**** could be decomposed to two matrices, **W** and **A**, where **W** = [**w**_1_, ⋯ , **w**_**N**_] has orthogonal columns that satisfy ${\text{w}}_{i}^{T}{\text{w}}_{j}=0\left(i,j=1,\cdots \;,N\right)$ for *i* ≠ *j*, and **A** is an upper triangular matrix with unit diagonal elements. The upper triangular matrix further multiplies the weight vector to construct an orthogonal weight vector, i.e., $\text{g}={\left[g_{1},\cdots \;,g_{N}\right]}^{T}=\text{A}\theta$ . Then, the integrated ROLS+D-opt learning algorithm performs a forward subset selection procedure from the full regression model, which is based on the following minimization criterion (Chen et al., 2003):

(4) $$\begin{array}{l} J_{CR}\left(\text{g},\lambda ,\beta \right)=J_{R}\left(\text{g},\lambda \right)+\beta \overset{N}{\underset{i=1}{\sum }}-\log\left({\text{w}}_{i}^{T}{\text{w}}_{i}\right)={\text{e}}^{T}\text{e}+\lambda {\text{g}}^{T}\text{g}+\\ \;\beta \overset{N}{\underset{i=1}{\sum }}-\log\left({\text{w}}_{i}^{T}{\text{w}}_{i}\right) \end{array}$$

where $J_{R}\left(\text{g},\lambda \right)={\text{e}}^{T}\text{e}+\lambda {\text{g}}^{T}\text{g}$ is the regularized error criterion, λ ≥ 0 is a regularization parameter, and β is a fixed small positive weighting for the D-optimality cost. The error reduction ratio is defined as:

(5) $$\begin{array}{l} {\left[crerr\right]}_{i}=\left(\left({\text{w}}_{i}^{T}{\text{w}}_{i}+\lambda \right)g_{i}^{2}+\beta \log\left({\text{w}}_{i}^{T}{\text{w}}_{i}\right)\right)/{\text{y}}^{T}\text{y} \end{array}$$

Based on the ratio in (5), significant regressors are selected in a forward-regression procedure, and the selection procedure is terminated when (Chen et al., 2003):

(6) $$\begin{array}{l} {\left[crerr\right]}_{l}\leq 0,for\;n_{s}+1\leq l\leq N \end{array}$$

#### Two-Level Learning Hierarchy RBF Network Learning Algorithm

In the integrated ROLS+D-opt learning algorithm, all candidate centers of the network are chosen from the input vectors of training samples, and the output weights θ_*i*_ in (1) can be obtained by linear learning algorithm (Chen et al., 2003). Therefore, only the uniform width ρ , regularization parameter λ , and D-optimality weighting parameter β need to be determined in the ROLS+D-opt algorithm. The selection of these three parameters has a great influence on the performance of the RBF network (Hong et al., 2003; Chen et al., 2009). A global optimization method is needed to determine the optimal combination of these three parameters.

**Figure 4:**
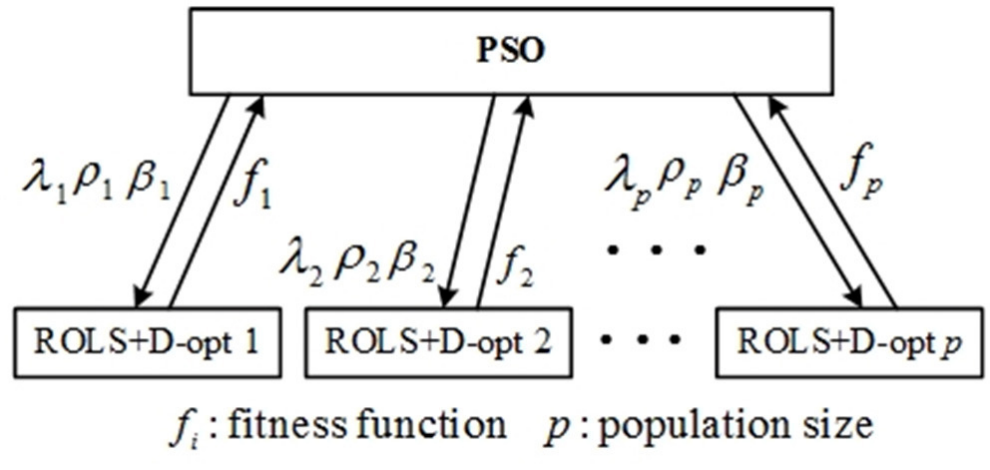
The architecture of two-level learning hierarchy in radial basis function networks.

A two-level learning hierarchy (TLLH) scheme is proposed by combining the PSO and ROLS+D-opt algorithms to train the RBF network, as shown in Figure 4. With the fitness function values given at the lower level, PSO (Kennedy and Eberhart, 1995; Shi and Eberhart, 1995) is used to learn the width ρ, regularization parameter λ, and D-optimality weighting parameter β of the integrated algorithm (ROLS+D-opt) at the upper level, while the lower level consists of *p* parallel integrated ROLS+D-opt learning algorithm for each set of parameters, [λ, ρ, β], provided by the PSO. *p* is the swarm size of the PSO, i.e., there are *p* particles in the PSO algorithm. PSO, like a swarm intelligent optimization method, has the characteristic of the parallel computation. In this study, all EEG samples are divided into a training set and a validation set. The *i*-th ROLS+D-opt algorithm constructs an RBF network using the training data set with a given particle [λ_*i*_, ρ_*i*_, β_*i*_], and the mean square error (MSE) over the validation set of the resulting RBF model is defined as the fitness function of the PSO algorithm:

(7) $$\begin{array}{l} \min f\left(K\right)=\frac{1}{n_{c}}\overset{n_{c}}{\underset{k=1}{\sum }}{\left(y\left(\text{x}\left(k\right)\right)-ỹ\left(\text{x}\left(k\right)\right)\right)}^{2} \end{array}$$

where *K* = [λ_*i*_, ρ_*i*_, β_*i*_] represents the particle, *y*(**x**(*k*) is the desired output of the validation sample, ỹ(**x**(*k*)) is the actual network output, and *n*_*c*_ is the size of the validation set. The smaller the fitness value, the better the generalization performance of the network (Chen et al., 1999, 2008).

The computational complexity of this TLLH scheme is determined by the total number of function evaluations at the upper level. Assuming that the swarm size of the PSO is *p*, the evolutionary generation is *T*, and the complexity of the ROLS+ D-opt algorithm is *C*_ROLS+D−opt_. Then, the complexity of the TLLH scheme is

(8) $$\begin{array}{l} C_{\text{TLLH}}=p\times T\times C_{\text{ROLS}+\text{D}-\text{opt}} \end{array}$$

since the PSO is only used to optimize three parameters of the integrated ROLS+D-opt learning algorithm, and the lower level presents a linear learning problem. The overall computational requirement of this scheme is much smaller than that of the scheme where a PSO is directly used to determine the RBF network structure as well as to learn all the network parameters (Billings and Zheng, 1995).

### Performance Evaluation

**Figure 5:**
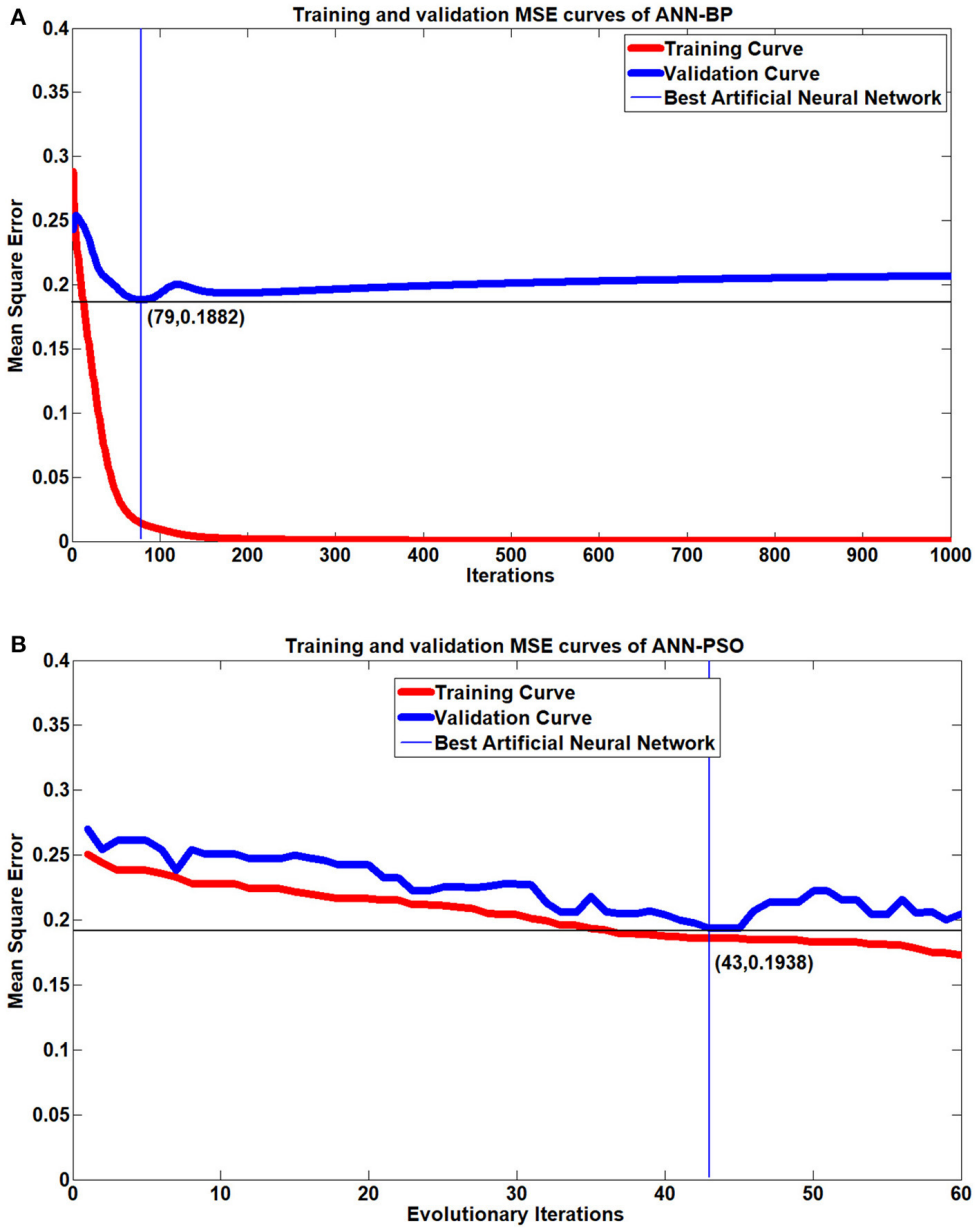
Mean square error (MSE) of the training and validation set for the early stopping of the classifier. **(A)** Training and validation MSE of artificial neural network (ANN)-back-propagation. **(B)** Training and validation MSE of ANN-particle swarm optimization.

To assess the performance of the proposed approach, the proposed RBF-TLLH was applied to the driving fatigue classification dataset and compared with the state-of-the-art neuron network models, including the RBF based on the ROLS+D-opt algorithm (RBF-ROLS+D-opt) (Chen et al., 2003), three-layer forward ANN with back-propagation (ANN-BP) (Zaw et al., 2019; Zhang and Pu, 2020), and three-layer forward ANN with PSO optimization (ANN-PSO) (Li and Liu, 2016). The RBF based on the ROLS+D-opt algorithm (RBF-ROLS+D-opt) has been widely used because of its robustness, sparsity of the parameters, and easy implementation (Chen et al., 2003). ANN-BP has the ability to approximate the non-linear function with arbitrary accuracy; therefore, it has been widely applied to various classification problems (Zaw et al., 2019; Zhang and Pu, 2020). The three-layer forward ANN with PSO optimization (ANN-PSO) is also widely used due to its advantages such as easy implementation, fewer adjustment parameters, and fast convergence (Li and Liu, 2016). The initial weights and thresholds are generated randomly within the interval [−1, 1] in the ANN-BP, the maximum epoch is set to 1,000, and the learning rate is 0.01. The MSE of the training data set is minimized as the objective function in the ANN-PSO. The variable parameters range is set to [−1, 1], the swarm size is set to 30, and the evolutionary iterations are set to 60. According to the empirical formula, the hidden nodes of these two ANN classifiers are both set to 30. In addition, in order to prevent over-fitting or over-training in the ANN network, a validation-based early stop strategy is used to select the best training parameters. Figure 5 shows the MSE curve of the training set and the validation set for classification. It can be seen that the best iteration number of the ANN-BP is 79, and the best iteration number of the ANN-PSO is 43, for this training result, according to the MSE curve of the validation set.

The regularization technique is employed in criterion function (4) in the proposed TLLH-RBF to prevent the over-fitting problem and improve the classification accuracy of the RBF network. The D-optimality cost is introduced to further enhance the efficiency and robustness of the selected subset model. The parameters, including the RBF width, regularization parameter, and D-optimality weighting parameter, are, respectively, set within range,ρ ∈ [1, 220], λ ∈ [10^−7^, 1], and β ∈ [10^−7^, 1], and optimized using the PSO. The swarm size *p* of PSO is set to 15, and the number of evolutionary iterations is set to 30. For further comparison, another RBF network classifier based on the ROLS+D-opt algorithm is also designed, where the width and D-optimality parameters are determined asρ = 110 and β = 10^−4^, respectively, by the trial-and-error method, whereas the regularization parameter λ is estimated by the Bayesian approach (MacKayi, 1992; Chen et al., 1996).

The classification results achieved by the four aforementioned neural network models were compared against each other. In all these classification models, when the actual output of the network is >0.5, the model classifies it as 1 (fatigue state); otherwise, the model classifies it as 0 (alert state). All samples are normalized firstly before the ANN is trained to prevent the ANN weights from being too large.

## Results

**Figure 6:**
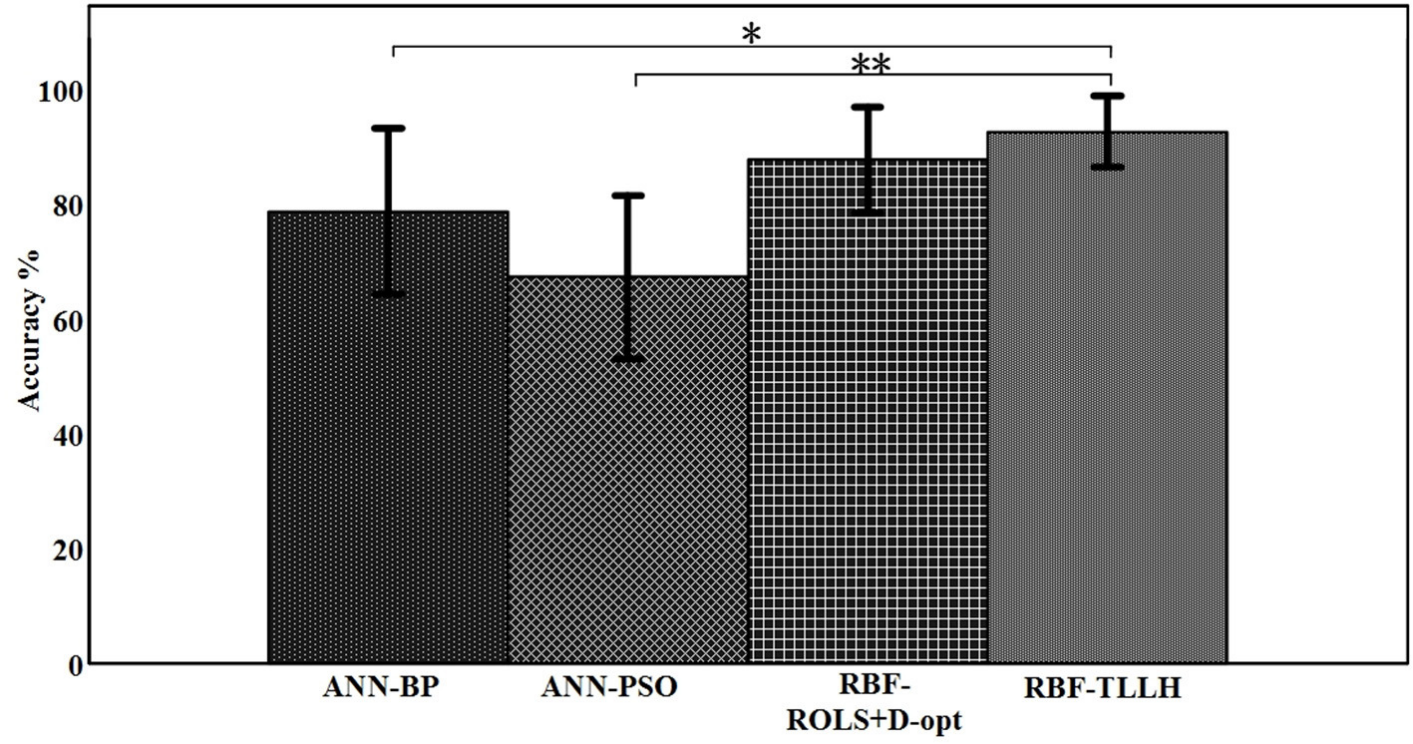
Bar chart of the mean accuracies when using different classifiers. *significantly different from control (*p* < 0.05); **significantly different from control (*p* < 0.005).

Table 1 summarizes the classification accuracy in driving fatigue detection as achieved by the four classification models using 6-fold cross-validation for each subject. The results show that the RBF-TLLH classifier achieves the highest accuracy for all the subjects in classifying the fatigue vs. alert states, with the mean value of 92.71 ± 6.26%. Overall, the ANN classifiers achieve lower classification accuracy than the RBF-based classifiers. Paired *t*-test was used for statistical comparison, as shown in Figure 6, showing that the proposed RBF-TLLH classifier significantly outperforms the other two ANN classifiers (*p* < 0.05) while the ROLS+D-opt-based RBF does not. Although no significant difference is observed between these two different RBF-based classifiers, the RBF-TLLH achieves higher accuracy and yields lower variance than the ROLS+D-opt RBF network, which that suggests the proposed RBF-TLLH is a more accurate and robust classifier in EEG driving fatigue detection in these two RBF-based classifiers.

**Table 1:** Average accuracy (%) of 6-fold cross-validation for each subject using different classifiers.

**Subjects classifier**	**Subject 1**	**Subject 2**	**Subject 3**	**Subject 4**	**Subject 5**	**Subject 6**	**Mean (SD)**
ANN-BP	75.00	59.58	90.42	95.42	78.33	62.08	76.81 (14.50)
ANN-PSO	57.50	54.58	86.67	84.17	60.00	60.83	67.29 (14.23)
RBF-(ROLS+D-opt)	82.08	77.92	97.08	98.75	91.67	79.17	87.78 (9.23)
RBF- TLLH	89.58	87.08	100.00	100.00	93.75	85.83	92.71 (6.26)

*SD, standard deviation*.

To further evaluate the classification performance of the proposed RBF-TLLH, six metrics including the true positive (TP), true negative (TN), false positive (FP), false negative (FN), specificity/true negative rate [TNR = TN/(TN + FP)], and sensitivity/true positive rate [TPR = TP/(TP + FN)] (Chai et al., 2017a,b), are computed from all subjects and summarized in Table 2. Compared to the ANN-BP and ANN-PSO models, the RBF-TLLH network model exhibits the best performance regardless of the specificity, sensitivity, and accuracy. In addition, the RBF-TLLH model significantly outperforms the ROLS+D-opt-based RBF model in sensitivity, demonstrating the superiority of the proposed approach to detect driving fatigue. Compared to the ROLS+D-opt-based RBF, the proposed RBF-TLLH model achieves a slightly lower specificity, but a much higher accuracy and sensitivity.

**Table 2:** Classification results of fatigue state *vs*. alert state for the validation set.

**Classification metrics**	**Classification model**			
	**ANN-BP**	**ANN-PSO**	**RBF-(ROLS+D-opt)**	**RBF-TLLH**
TP	552	517	576	665
TN	554	452	688	670
FN	168	203	144	55
FP	166	268	32	50
Sensitivity/TPR (%)	76.67%	71.81%	80.00%	92.36%
Specificity/TNR (%)	76.94%	62.78%	95.56%	93.06%
Accuracy (%)	76.81%	67.29%	87.78%	92.71%

True represents the fatigue state; false represents the alert state.

*TP, true positive; TN, true negative; FN, false negative; FP, false positive*.

**Figure 7:**
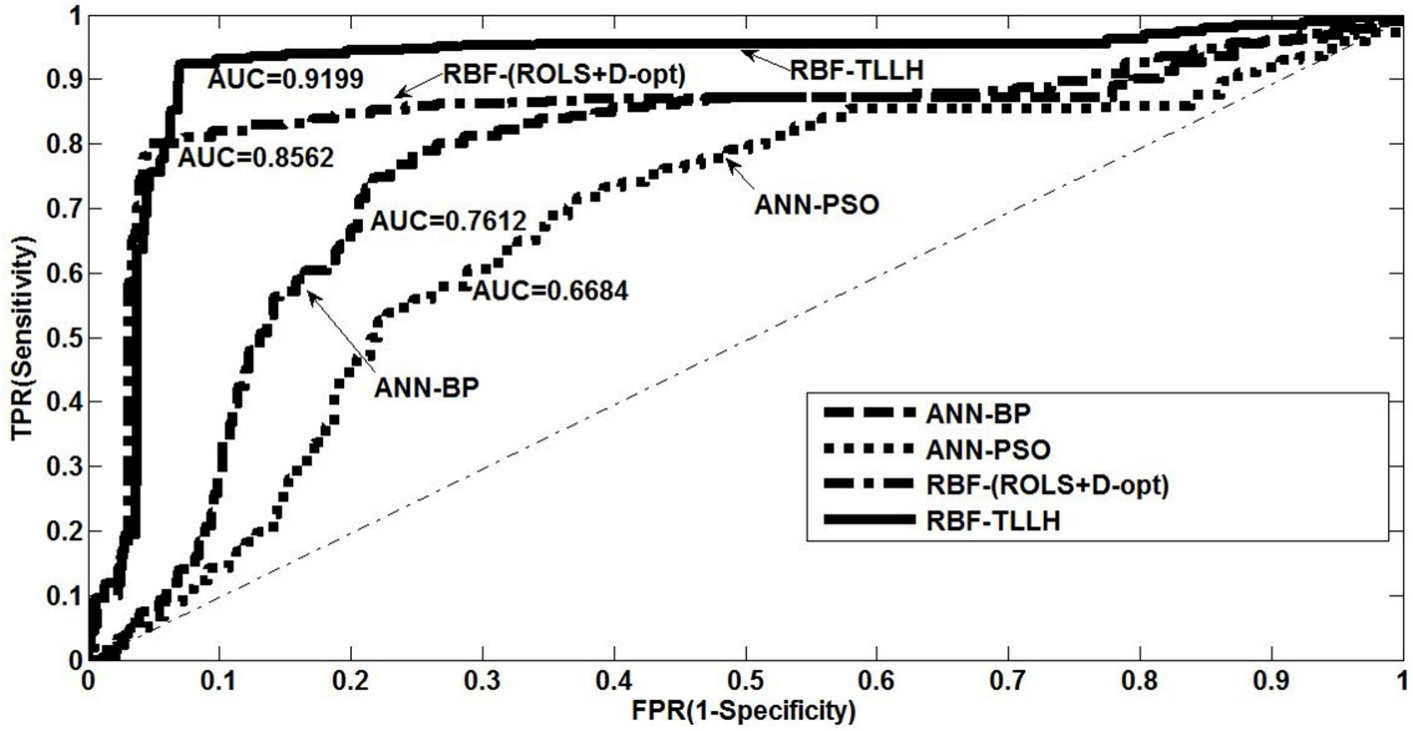
Receiver operating characteristic plot with area under the curve values for different classification models: (1) artificial neural network (ANN) using back-propagation algorithm, (2) ANN using particle swarm optimization, (3) radial basis function (RBF) network using ROLS+D-opt algorithm, and (4) RBF network using two-level learning hierarchy method.

The receiver operating characteristic (ROC) curve analysis is also conducted, and the results are summarized in Figure 7. The ROC curve is a plot of TPR vs. false-positive rate (FPR/1-specificity) by varying different threshold ratios as a sweeping variable. A random classification model is expected to show a straight line connecting (0, 0) to (1, 1) (diagonal dash–dot line in Figure 7). Any ROC curve located in the lower-right triangle indicates that the classifier is worse than random guessing, while the ROC curve that lies in the upper-left triangle indicates that the model performs better than random guessing (Fawcett, 2006; Chai et al., 2017b). The area under the curve (AUC) of the ROC curve is then calculated to evaluate the model performance. As shown in Figure 7, the proposed RBF-TLLH achieves the best upper-left ROC curve and yields the highest AUC value (0.9199) among all classifiers, demonstrating the best performance in the detection of driving fatigue.

## Discussion

EEG has the advantages of non-invasiveness and high temporal resolution for brain activity measurement and has been widely considered as a good indicator of the transition between the alert and fatigue states. Power spectral density (PSD), which converts the time domain of EEG data into the frequency domain, has been widely employed in traditional EEG-based fatigue detection studies. EEG signals can then be generally divided into five bands, i.e., Delta (0.5–4 Hz), Theta (4–8 Hz), Alpha (8–13 Hz), Beta (13–30 Hz), and Gamma (30–42 Hz) waves, according to frequency and amplitude characteristics (Sikander and Anwar, 2019). It has been found that the increase of EEG alpha band spindles is associated with the fatigue state when participants drive in the actual monotonous driving environment (Simon et al., 2011). It has also been demonstrated that EEG is sensitive to fluctuations in vigilance and has been shown to predict performance degradation due to sustained mental workload. During the monotonous driving task, the EEG alpha bursts will be dominant in the central and posterior EEG channels, which is a signal of drowsiness and reduced vigilance (Simon et al., 2011).

**Figure 8:**
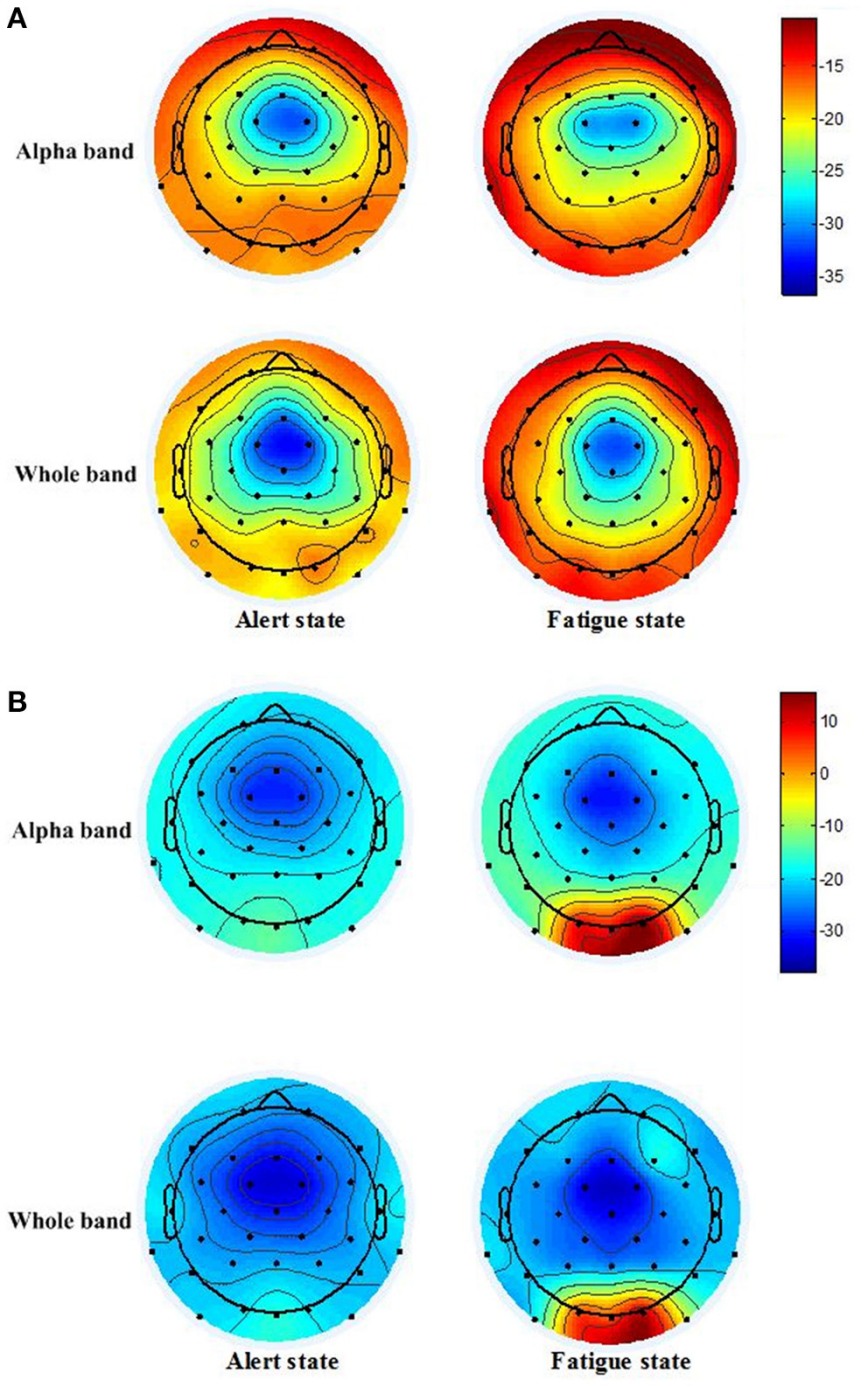
Power spectral density (PSD) distributions of EEG signals for subjects 1 and 3. **(A)** PSD distributions of the alpha band of EEG signals in the alert state (a1) and in the fatigue state (a2). PSD distributions of the whole band of EEG signals in the alert state (a3) and in the fatigue state (a4) for subject 1. **(B)** PSD distributions of the alpha band of EEG signals in the alert state (b1) and in the fatigue state (b2). PSD distributions of the whole band of EEG signals in the alert state (b3) and in the fatigue state (b4) for subject 3.

Taking subject 1 and subject 3 as examples, Figure 8 shows the PSD distributions of the alpha (8–13 Hz) and whole (1–45 Hz) wave bands of the two states (alert and fatigue), respectively. It can be observed that the PSD distributions between the alert and fatigue states show an apparent characteristic difference. The PSD difference between the alert and fatigue states of subject 3 is also more significant compared with the PSD distributions of subject 1. This is consistent with the results in Table 1 when using the RBF-TLLH classifier, that is, the average classification accuracy achieved in subject 3 (100%) is higher than that achieved in subject 1 (89.58%). In addition, regarding the PSD distributions of the subjects during the fatigue state, the alpha band of EEG signals carries the majority of the information among the whole PSD distributions. These findings validate that the EEG has a distinct difference in characteristics between the alert and fatigue states, demonstrating the feasibility of using EEG as an effective approach to detect driving fatigue.

Although EEG signals provide distinct characteristics associated with between the alert and fatigue brain states, it is still necessary and critical to develop a high-performance classifier in order to monitor the brain state alteration during driving. Studies have demonstrated that the RBF neural network is a promising classifier due to its linear-in-the-parameters network structure, strong non-linear approximation ability, and desired generalization property (Li Y. et al., 2017; Bose et al., 2019; Li et al., 2019). The performance of the RBF network heavily relies on the number of hidden nodes, center vectors, and output weights. These parameters can be learned using some global optimization methods; however, direct optimizing of all the parameters using the global optimization algorithm is hampered by the high evaluation cost and slow convergence. This study aims to enhance the performance of the EEG-based driving fatigue classification model using a two-level learning hierarchy RBF network (RBF-TLLH). The RBF-TLLH is constructed by integrating the ROLS+D-opt algorithm, which combines the regularized orthogonal least squares and D-optimality experimental design at the lower level and the PSO at the upper level.

At the lower level of the RBF-TLLH, the ROLS+D-opt learning algorithm is employed. With the ROLS+D-opt learning algorithm, all the candidate centers of the RBF network are chosen from the input vectors of the training samples, and the output weights in (1) can be obtained by linear learning algorithm (Chen et al., 2003). Moreover, the entire RBF network model construction procedure is terminated automatically when condition (6) is reached. Therefore, there are only three parameters left in the ROLS+D-opt algorithm, i.e., the uniform width, the regularization parameter, and the D-optimality weighting parameter, to be determined. At the upper level of the RBF-TLLH, PSO is employed. PSO is typically characterized as an algorithm with a simple concept, easy implementation, and good computational efficiency (Kennedy and Eberhart, 1995; Shi and Eberhart, 1995). As a swarm intelligent optimization method, PSO has the characteristic of parallel computation. Therefore, PSO is employed to optimize the three core parameters of the ROLS+D-opt algorithm at the upper level, while the ROLS+D-opt algorithm automatically constructs RBF networks at the lower level to enhance the classification performance. As shown in Tables 1, 2, the RBF network obtained from the proposed learning hierarchy has demonstrated its superior performance with a mean classification accuracy of 92.71% and an AUC-ROC value of 0.9199 against other methods, making it a promising candidate for driving fatigue detection in the future.

Experiment EEG data were collected in six healthy subjects in a simulated driving environment and were utilized to evaluate the performance of the proposed RBF-TLLH algorithm by comparing it against three other classifiers. The results show that the proposed RBF-TLLH achieves a substantial increase in classification accuracy compared to other approaches. Particularly, the sensitivity of the proposed RBF-TLLH model is much higher than the other three methods. The high sensitivity performance suggests that the RBF-TLLH-based driving fatigue detection system is more sensitive in detecting fatigue states, which is critical to ensure safe driving.

It is noteworthy that, prior to the classification using the RBF-TLLH method, PCA is necessarily adopted to alleviate the high dimension problem of multi-channel EEG signals. In this study, the PCA method was applied for dimensionality reduction of the EEG signals, and the first 10 PCs of each channel were selected to obtain better driving fatigue detection power. Apparently, the number of preserved PCs would affect the performance of the feature extraction, which would further affect the performance of the driving fatigue detection model. This number of preserved PCs is determined based on multiple trial calculations to ensure that the preserved PCs account for over 80% of the total variance of the original signals.

The classification performance of the neural network is directly related to its network structure and weights. For the ANN classifier, the three-layer forward ANN is formed with 320 input nodes, 30 hidden nodes, and one output node, yielding 9,661 weight and threshold parameters to be optimized. It is generally very difficult to deal with such a high-dimension optimization problem for the back-propagation (BP) algorithm mainly because the gradient-based BP algorithm is sensitive to the initial parameters and easily trapped in the problem of local minima (McLoone et al., 1998). The premature and stagnation phenomenon will also occur during the later stage of evolution when the PSO solves this complex problem. These shortcomings eventually result in the poor performance of the ANN-based classifier for classifying driving fatigue.

Computation efficiency is also evaluated in this study to test the application feasibility of the proposed RBF-TLLH classifier in real-time driving fatigue detection. During the operation process of the real-time classification, based on the obtained features and the parameters of the RBF network for the detection system, the classifier can rapidly determine the driving fatigue detection result through Equation (1). The testing results show that the execution time is only about 0.011 s in a MATLAB environment [an Intel(R) Core(TM) i7-4500U CPU@ 1.8 GHz, 8 GB RAM]. This is because there is no necessity to train the classifier again for the operation of real-time classification. Specifically, with the saved parameters and particular features, the classifier only needs to compute the feedforward neural network function based on (1) for classification system, which can take <0.1 s when developed in C language.

Despite the improvements achieved in this study, there are limitations that can be addressed in future studies. Only six subjects participated in the simulated driving fatigue experiment, so the sample size of this study is small. Our future efforts will be devoted to collecting a larger sample size from either simulated or real driving fatigue test to further evaluate the performance of the proposed RBF-TLLH in detecting driving fatigue. Furthermore, deep neural network models have attracted increasing attention in recent years because of their powerful non-linear fitting capability, high dimensional data processing capability, large fault tolerance, and strong feature extraction capability. The proposed RBF-TLLH will be compared with deep neural network models, such as LSTM, to further evaluate its performance in driving fatigue detection. In addition, recent research on latent analysis have proved its power in feature extraction, and this method will be used in a future study (Wu et al., 2019, 2020). Lastly, labeling the driving states for a larger sample size could be expensive and time-consuming. In order to solve this problem, semi-supervised classification algorithms will be considered in the future work (She et al., 2018, 2019, 2020a,b; Wu et al., 2018a,b).

## Conclusion

In this study, a two-level learning hierarchy RBF network has been developed for EEG-based driving fatigue detection to optimize the classification performance (fatigue vs. alert). The experimental results show that the proposed method achieved a superior classification performance compared to other methods in terms of prediction accuracy and computational efficiency. Due to the significantly fewer core parameters to be determined for training the RBF classifier, this proposed approach presents excellent ease of use and large potential application possibilities for the detection of driving fatigue in the future.

## Data Availability Statement

The raw data supporting the conclusions of this article will be made available by the authors, without undue reservation.

## Ethics Statement

The studies involving human participants were reviewed and approved by Guangdong Provincial Work Injury Rehabilitation Hospital. The patients/participants provided their written informed consent to participate in this study.

## Author Contributions

ZR conceived this study and contributed to the experimental design. ZR performed the computational analysis with the assistance of BC and HZ. CW and YM contributed to subject recruitment and data collection. ZR, RL, YL, and YZ analyzed the results and prepared the manuscript. All the authors reviewed the results and approved the final manuscript.

## Conflict of Interest

The authors declare that the research was conducted in the absence of any commercial or financial relationships that could be construed as a potential conflict of interest.
